# Development of Sustainable High Performance Epoxy Thermosets for Aerospace and Space Applications

**DOI:** 10.3390/polym14245473

**Published:** 2022-12-14

**Authors:** Roxana Dinu, Ugo Lafont, Olivier Damiano, Alice Mija

**Affiliations:** 1 Université Côte d’Azur, Institut de Chimie de Nice, 06108 Nice, France; 2 ESA, ESTEC, Keplerlaan 1, P.O. Box 299, NL-2200 AG Noordwijk, The Netherlands; 3 Thales Alenia Space, 5 Allée des Gabians, 06156 Cannes la Bocca, France

**Keywords:** epoxy, thermoset, high performance, high glass transition, space and aerospace applications

## Abstract

There is an imperative need to find sustainable ways to produce bisphenol A free, high performance thermosets for specific applications such as the space or aerospace areas. In this study, an aromatic tris epoxide, the tris(4-hydroxyphenyl)methane triglycidyl ether (THPMTGE), was selected to generate high crosslinked networks by its copolymerization with anhydrides. Indeed, the prepared thermosets show a gel content (*GC*) ~99.9% and glass transition values ranged between 167–196 °C. The thermo-mechanical properties examined by DMA analyses reveal the development of very hard materials with *E*′ ~3–3.5 GPa. The thermosets’ rigidity was confirmed by Young’s moduli values which ranged between 1.25–1.31 GPa, an elongation at break of about 4–5%, and a tensile stress of ~35–45 MPa. The TGA analyses highlight a very good thermal stability, superior to 340 °C. The Limit Oxygen Index (*LOI*) parameter was also evaluated, showing the development of new materials with good flame retardancy properties.

## 1. Introduction

The ever-increasing progress of humanity has led to the emergence of new engineering challenges and demands. Due to their many unique properties such as light weight, low cost and easy processing, plastics became the main substitute for conventional materials. For example, world plastic consumption in 1970 was about 30 million tons, growing exponentially with the population needs, reaching about 400 million tons in 2020 [1]. In the engineering fields, thermoset materials such as phenolic and urea formaldehyde resins, unsaturated polyesters, or epoxy resins, are used intensely due to their excellent properties resulting from the high crosslinked tridimensional structures [2]. Characteristics such as high thermal and mechanical properties, good processability, compatibility and chemical resistance with other polymers, good creep resistance and low shrinkage, make the epoxydic resins the most used class of materials from the thermosets category [2,3,4,5]. Thus, epoxy resins are widely used as coatings, composite matrices, adhesives for electronic components, structural materials in construction, and semiconductor capsules for applications in the automotive, naval, aerospace and space industries [2,4,5,6,7].

At the industrial level, the greater part of the epoxy resins used are made from diglycidyl ether of bisphenol A (DGEBA) which is obtained by the reaction between bisphenol A (BPA) and epichlorohydrin [7]. According to REACH regulations, BPA has endocrine disrupting properties and thus appears to be an estrogenic receptor antagonist, being classified as a carcinogen, mutagen and reprotoxic (CMR) [8,9,10]. Awareness of the BPA toxicity has led to the restriction of its use in numerous countries, thus generating an industrial crisis and an imminent need to find alternatives to epoxy resins that meet and provide characteristics similar or even better to those generated by DGEBA. Recently, we reported the synthesis of bis-epoxy and tris-epoxy biobased monomers derived from vanillin (DGEVA) and phloroglucinol; monomers there were further crosslinked with methyl nadic anhydride and hexahydro-4-methylphthalic anhydride. These materials have a biobased organic carbon ~70%. The synthesized thermosets show high performances, as *E*′ modulus ~3 GPa, glass transition values ~96–108 °C for DGEVA based thermosets and 190–210 °C for TGPh based ones together with a low hydrophilicity, a high gel content ~99.9%, high thermal stability, etc. [11].

In contrast with these reported studies, we address the answer to monomers availability from an industrial point of view. As we know very well, there is currently a very strong need to find solutions and alternatives, especially with regard to the problem of resource depletion for industry. As we demonstrated recently, biobased solutions are working very well. But if we need to manufacture materials used in different industries from everyday applications to aerospace or space, access to cheap and available raw materials is essential. The main purpose of this work was to develop thermoset epoxy resins with a commercial monomer that is non-toxic, easily available, and cheap, while the products are marketable, easy, and fast to manufacture, with high-end properties and high added value. The tris(4-hydroxyphenyl)methane triglycidyl ether (THPMTGE) was selected as a monomer as it is commercialized at industrial scale and its synthesis could be done from biobased synthons (condensation of phenol with benzaldehyde followed by glycidylation), [12,13,14,15,16,17]. In addition, in comparison with the previous work [11], the THPMTGE monomer has the advantage of containing a tri-aromatic core, differing from DGEVA and TGPh, which have mono-aromatic cores.

The THPMTGE is currently used in applications that come into contact with the human body such as cosmetics, or in daily applications such as paints, coatings, inks for printing or writing, etc. To the best of our knowledge, the studies regarding the THPMTGE used to develop polymer-based materials remain very modest [18,19,20,21,22]. Khalil et al. [19] investigated three epoxy systems: epichlorohydrin (EP), trimethylolpropane triglycidyl ether (TMPTGE), and tris(4-hydroxyphenyl)methane triglycidyl ether (THPMTGE) to incorporate *β*-cyclodextrin into electrospun polymeric nanofibers for the removal of steroid hormone micropollutants. The obtained materials containing β-cyclodextrin showed the ability of these materials to be used as composite nanofiber membranes with enhanced estradiol removal of about 95% and high-water permeability. El Gazzani et al. [21] used the triglycidyl ether of tris(4-hydroxyphenyl)methane (THPMTGE) for the development of high temperature epoxy expanded material as substitution of phenolic resins. Chung et al. [22] crosslinked THPMTGE with methacrylic acid in the presence of 4-dimethyl aminopyridine to obtain a new polyester for the development of composite resins with reduced curing shrinkage. These thermosets revealed good properties and characteristics with potential applications as photocurable dental materials.

This work aims to design sustainable, bisphenol A free, high performance thermoset resins tailored to meet industrial manufacturing requirements through an easy and rapid processing with reduced preparation steps, without high energy consumption in terms of temperatures or pressure, and by using cost competitive and commercially available feedstock. For this purpose, we choose to crosslink tris(4-hydroxyphenyl) methane triglycidyl ether (THPMTGE) with four phthalic anhydride derivatives: 1,2-cyclohexane dicarboxylic anhydride, hexahydro-4-methyl phthalic anhydride, methyl nadic anhydride, and cis-1,2,3,6- tetrahydro phthalic anhydride. These anhydrides were selected due to their reactivity but especially because some of these derivatives of phthalic anhydride are actually biobased. Their synthesis from bio-sourced feedstock is studied extensively. For example, the 3-methylphthalic anhydride (bio-MPA) is currently produced from biomass-derived furan and maleic anhydride [23,24,25]. Also, these four anhydrides are used industrially in the development of a wide range of items such as personal care products, paints, coatings, ink and colorant products, surface treating, greases, and many others. Similarly, the crosslinking reactions were initiated by 2,4,6-tris(dimethylaminomethyl)phenol, a compound currently used in formulations of drugs, textiles, personal care products (cosmetics, tattoo inks, hair dye), food colorants, inks, food packaging, paper plates, cutlery, etc. The physico-chemical and thermo-mechanical properties of the developed polyester-based thermosets in the present study were investigated by various analyses such as differential scanning calorimetry (DSC), attenuated total reflection Fourier transform infrared (ATR-FTIR) spectroscopy, thermogravimetric analysis (TGA), dynamic mechanical analysis (DMA), tensile tests, Shore hardness tests, water absorption and gel content.

## 2. Materials and Methods

### 2.1. Materials

**Figure 1:**
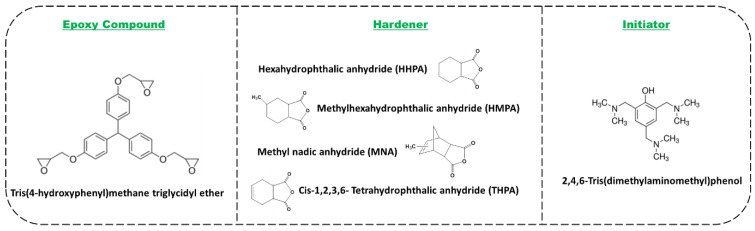
Molecular structures of the used compounds.

Thermoset resins were designed starting from a commercially available epoxy compound such as tris(4-hydroxyphenyl)methane triglycidyl ether, THPMTGE (annotated with “T”). The used crosslinking agents were four anhydrides: *cis*-1,2-cyclohexanedicarboxylic anhydride (HHPA; 95%), hexahydro-4-methylphthalic anhydride (HMPA; 96%), methyl nadic anhydride (MNA; ≥95%) and *cis*-1,2,3,6-tetrahydro phthalic anhydride (THPA; 95%). The crosslinking reactions were initiated by 2,4,6-tris(dimethylaminomethyl)phenol (“D”; 95%). All the chemical compounds were supplied from Merck, Sigma Aldrich (France), and used without any further purification. The molecular structures of these compounds are given in Figure 1. 

### 2.2. Thermosetting Resins Manufacturing

The proper amount of epoxy monomer, THPMTGE (annotated with “T”), was placed on a heating plate at 50 °C to decrease its viscosity and later mixed with the established quantity of anhydride comonomer. The copolymerization formulations were developed using a stoichiometric ratio of the anhydride [A] to epoxy groups [E] defined by R = 1 [26,27], the amount of each component used for the development of 100 g of resin being depicted in Table 1. 2,4,6-tris(dimethylaminomethyl)phenol (abbreviated with “D”) was used as initiator and after a preliminary study in which different proportions (0.5 wt.%, 1 wt.% and 2 wt.%; in Figure S1 example of T-MNA curing) were tested, it was found that the addition of 1% based on the total weight of the epoxy mixture provided the best crosslinking parameters in terms of reaction enthalpy and temperature interval. 

The formulations of epoxy systems have been coded for an easier identification. For example, the formulation with THPMTGE and HHPA and initiator was coded with “T-HHPA”. The freshly prepared mixtures were poured in silicon molds and cured in an oven at 150 °C for 3 h and then post-cured for 2 h at 180 °C to complete the reaction. This program was previously determined by the DSC analysis of thermal curing parameters.

### 2.3. Experimental Techniques

#### 2.3.1. Differential Scanning Calorimetry (DSC)

The copolymerization reactions between the tris-epoxy and anhydride comonomers were followed by differential scanning calorimetry (DSC) analyses with a Mettler Toledo DSC 3 apparatus and STAR Software. Freshly prepared formulation samples (8–10 mg) were submitted to dynamic heating from 25 °C to 250 °C, applying a heating rate of 10 °C·min^−1^ under a flow of 50 mL·min^−1^ nitrogen. The reaction parameters such as the temperature interval (*T*_onset_-*T*_end_), enthalpy (Δ*H*), and maximum peak temperature (*T*_peak_) were evaluated. The secondary transitions (α relaxations or so-called glass transitions, *T*_g_) of the designed thermosets were determined by analyzing the cured samples in two heating/cooling cycles from 0 °C to 260 °C at a rate of 20 °C·min^−1^. The glass transition value was estimated as the inflection point of the DSC curve in the second heating scan.

#### 2.3.2. Attenuated Total Reflection Fourier Transform Infrared (ATR-FTIR) Spectroscopy

FTIR spectroscopy was used to analyze the structure of the raw materials and final thermosets. IR spectra were recorded at room temperature, between 4000 and 500 cm^−1^, with 64 scans and a resolution of 4 cm^−1^ employing a Nicolet iS50 FT-IR spectrometer equipped with a GladiATR (PIKE Technologies, Inc.) single diamond attenuated total reflectance. The background was automatically subtracted. The structural evolution during the fresh mixture heating was followed under a dynamic heating ramp from 30 °C to 250 °C at a heating rate of 10 °C·min^−1^. During these experiments, IR spectra were collected every 2 min.

#### 2.3.3. Thermoset’s Density

The volume of rectangular specimens (50 × 8 × 4 mm^3^) from each formulation was firstly calculated, and then their mass was determined with a Mettler Toledo ML3002T precision balance. The thermosets’ density was calculated using the ratio of mass to volume, and the final values are the average of four samples.

#### 2.3.4. Dynamic Mechanical Analysis (DMA)

The thermo-mechanical behaviour of polyester thermosets was analyzed by using a Mettler-Toledo DMA 1 instrument equipped with STAR software. Parameters such as storage (*E*′) and loss (*E*″) moduli and the damping factor (tan δ = *E*″/*E*′) were obtained by examining three thermoset rectangular samples for each system using a three-points bending fixture under a dynamic heating from −70 °C to 300 °C at 3 °C·min^−1^ at a frequency of 1.0 Hz and an amplitude of 20 µm.

#### 2.3.5. Shore Hardness Tests

The hardness of the materials was determined utilizing a Zwick Roell 3116 hardness tester by measuring the penetration depth of an analog Shore D device applying a loading force of 50 N. For a greater accuracy of the analysis, each formulation was tested five times, the hardness value being determined as their average. The test method used to measure the Shore D hardness of thermosets was in accordance with ISO 7619-1, ASTM D2240 and ISO 868 standards.

#### 2.3.6. Tensile Testing

The mechanical properties (e.g., Young’s modulus, tensile strength, tensile stress, etc.) of the designed thermosets were investigated by tensile testing according to the ASTM D638-08 and ISO 527-1 standards. Five dumbbell specimens (30 mm effective length, 5 mm width and 3 mm thickness) for each system were tested at a crosshead speed of 10 mm·min^−1^ until breaking using an Instron 34SC-5 instrument operated by BlueHill software (Instron, Norwood, MA, USA).

#### 2.3.7. Thermogravimetric Analysis (TGA)

The thermal stability of the thermosets was investigated under oxidative (air) and inert (N_2_) atmosphere (50 mL·min^−1^) with a Mettler Toledo TGA 2 and STAR Software. Crosslinked samples (~10–12 mg) were tested during heating from 25 to 1000 °C, in alumina crucibles, applying a heating rate of 10 °C·min^−1^. The degradation temperature of materials was considered as at the temperature at which the systems lost 5% of their mass (*T*_5%_). Thermal stability was investigated two times for each system.

#### 2.3.8. Water Absorption (*WA*%)

The materials’ capacity to absorb water was studied in accordance with the ASTM D570 standard test method, where the three conditioned samples for each system (at 50 °C for 24 h) were immersed in distilled water at room temperature. At each 24 h, the thermoset samples were removed from water, wiped with filter paper, and then weighed. The water absorption percentage of the epoxy systems was calculated with the equation [28]:

(1) $$WA,\;\%=\frac{w_{d}-w_{0}}{w_{0}}\times 100$$

where *w*_0_ represents the conditioned initial mass, while *w*_d_ is the wet mass of the sample. The water absorption percentage value was recorded after 24 h of immersion and at saturation level.

#### 2.3.9. Gel Content (*GC*)

The solvent extraction method was used for the thermosets’ gel content (*GC*) analysis. Three rectangular samples from each system (10 × 10 × 5 mm^3^) were first conditioned in an oven at 50 °C for 48 h, and then their initial mass (*w*_0_) was determined using a Mettler Toledo ML3002T precision balance. After weighing, the samples were completely immersed in 5 mL of toluene and maintained at room temperature for 48 h. At the end of the test, samples were extracted from the solvent, dried at 50 °C for 24 h and then weighed (*w*_f_). Based on the initial and final mass of the studied thermosets, their gel content was determined with equation [29]:

(2) $$\mathrm{GC},\;\%=100-\left[\frac{\left(w_{0}-w_{f}\right)\times 100\;}{w_{0}}\right]$$

## 3. Results and Discussions

### 3.1. Crosslinking Behavior of the Epoxy-Based Thermosetting Formulations

#### 3.1.1. Differential Scanning Calorimetry (DSC) Investigation

**Figure 2:**
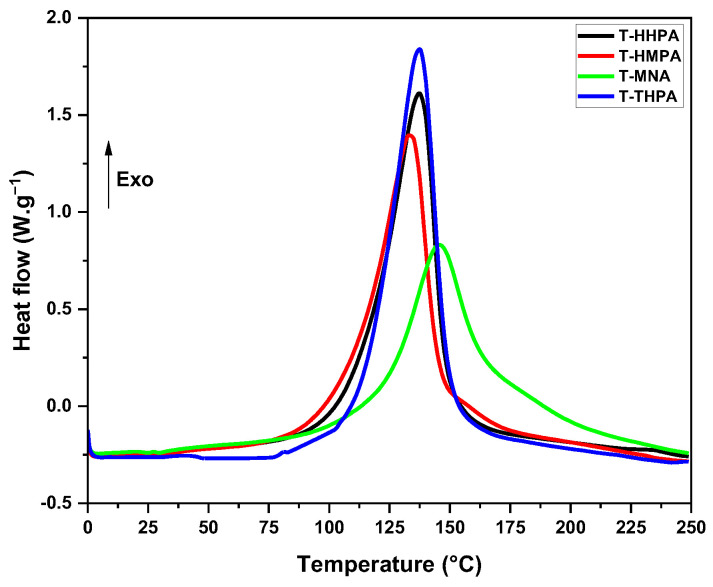
Dynamic DSC curves of the epoxy-anhydride crosslinking formulations under heating at 10 °C·min^−1^.

To investigate the thermodynamic aspects of the epoxy-anhydride crosslinking reaction, the evolution of the heat flow in function of the temperature was analyzed (Figure 2). The obtained reactions parameters: reaction enthalpy (Δ*H*), the temperature interval (*T*_onset_-*T*_end_), and the maximum reaction peak temperature (*T*_peak_) were summarized in Table 2. 

As can be seen from Figure 2, the cure profile of the four systems seems to be similar, except for the system crosslinked with MNA anhydride, which has a different molecular arrangement than the other three anhydrides with more similar structures. In the heat flow evolution with temperature, we can highlight that the formulations of THPMTGE with HHPA, HMPA and THPA anhydrides are reacting with a narrow and well-defined exothermic peak, while in the system with MNA anhydride the exothermic peak is reduced in intensity and is wider.

The onset reaction temperature of MNA-formulation is about ~95 °C, reaching a maximum of reactivity at around ~146 °C, and then completing the reaction at *T* > 240 °C. For the formulations of THPMTGE with the derivatives of phthalic anhydride (HHPA, HMPA and THPA), the crosslinking reactions start at lower temperature, ranging between 55–75 °C, with a maximum at around ~134–138 °C, the exothermic event returning at a quasi-linear response at around ~180–200 °C. Knowing that the lower the *T*_peak_ value, the higher the reactivity [30], in analyzing the obtained data it can be seen that the highest reactivity was obtained for the T-HMPA formulation, followed by those with HHPA and THPA anhydrides, while the lowest reactivity was found for the system T-MNA.

Integrating the area under the exothermic event, the reaction enthalpy (Δ*H*) of the four formulations was evaluated and the obtained values are given in Table 2. All the four anhydrides provide very good reactivity with enthalpy of reaction values superior to 280 J·g^−1^, the maximum being obtained for T- HHPA formulation with ΔH ~340 J·g^−1^. 

The efficiency of the curing reaction of all the epoxy-based systems was investigated by the solvent extraction method and the estimation of networks gel content (*GC*%). The thermosets *GC*% values were calculated after 48 h of immersion in toluene. The obtained data (Table 2) show values greater than 99.9%, confirming the efficiency of THPMTGE- anhydride crosslinking reactions being superior to the TGPh (*GC* = 99.6–99.8%) and DGEVA (*GC* = 94.8–99.2%) formulations designed in a previous study [11].

#### 3.1.2. Attenuated Total Reflection Fourier Transform Infrared (ATR-FTIR) Spectroscopy

ATR-FTIR spectroscopy was used as a complement to the DSC analyses to follow the chemical structural modifications when the fresh formulations are submitted to a heating program, in situ, such as that used by DSC. The FTIR spectra of the initial components but also of the cured and post-cured thermoset resins were then recorded at room temperature, and the obtained spectra are displayed in Figures S3 and S4, respectively.

In the FTIR spectrum of THPMTGE epoxy monomer, the principal signals are represented by the asymmetric stretching vibration for the epoxy ring at 912 cm^−1^ (C–O st.) and 828 cm^−1^ (C–O–C st.). Another significant signal appears at 1027 cm^−1^ corresponding to the CH_2_–O–CH_2_ asymmetric stretching vibrations of the ether groups. The aromatic rings’ signals appear by the peaks at 1505 cm^−1^ characteristic to the C–C st. vibrations and at 1607 cm^−1^ for the C=C st. vibrations, while the absorption band at 2873–2998 cm^−1^ corresponds to the C–H symmetric and asymmetric stretching vibrations. 

**Figure 3:**
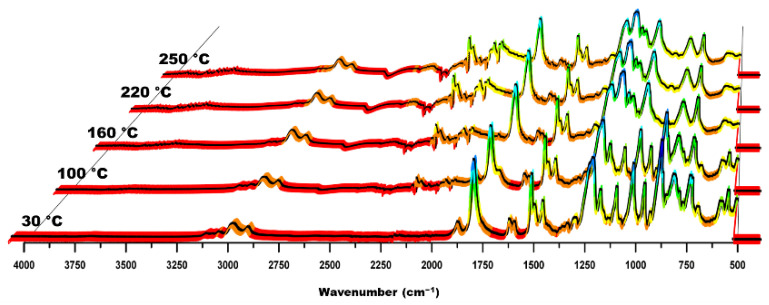
FTIR spectra registered during heating the T-HHPA formulation. Temperatures are indicated on each spectrum.

**Figure 4:**
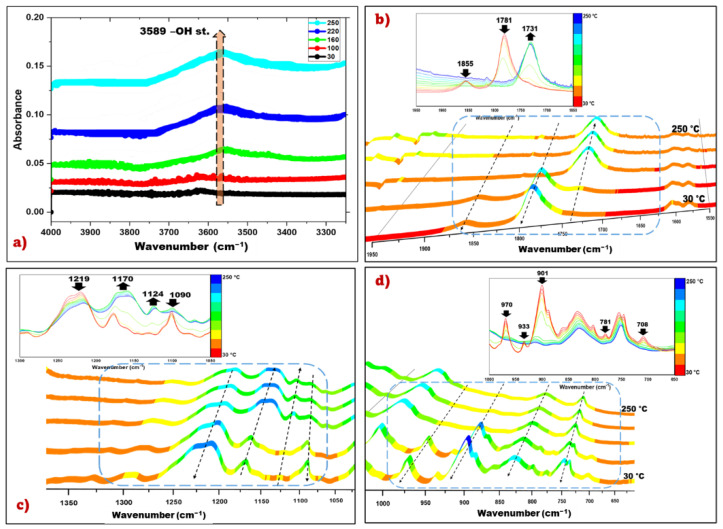
FTIR spectra in function of temperature T-HHPA system in (**a**) 4000–3300 cm^−1^ region; (**b**) 1950–1550 cm^−1^ region; (**c**) 1350–1050 cm^−1^ region and (**d**) 1000–650 cm^−1^ region.

The evolution of the chemical structure during the curing process was also investigate by FTIR. A fresh sample was heated from 30 to 250 °C at a heating rate of 10 °C·min^−1^, and the IR spectra was recorded every 2 min. In Figure 3, the FTIR spectra registered during heating for T-HHPA formulation are displayed. Analyzing the FTIR spectra, we can observe a clear evolution and change in four main areas, their graphical representations being present in Figure 4. 

During epoxy-anhydride crosslinking through polyesterification and/or polyetherification reactions, –OH groups may be produced when using DMP 30 as an initiator [31,32,33,34,35]. As can be seen in Figure 4a, the stretching vibration peak of –OH found at 3589 cm^−1^ increased in intensity with increasing temperature, a phenomenon attributed to the generation of hydroxyl groups during the ring opening reaction of the epoxy group. In Figure 4b can be seen the formation of ester groups by the appearance of the carbonyl peak (C=O stretching vibration of ester group) at a wavenumber of 1731 cm^−1^. In addition, in the thermoset samples, the peaks at 1855 cm^−1^ and 1781 cm^−1^ (carbonyl groups in HHPA) disappeared completely, indicating that the anhydride groups had fully reacted [36,37]. The increase of the absorption bands at 1170 cm^−1^ and 1124 cm^−1^ (Figure 4c) which characterize the C–O stretching vibration of the ester group indicate the esterification reactions through the crosslinking of epoxy compound with the anhydrides [31]. 

The evolution of the absorption band of the epoxy groups as a function of temperature is represented in Figure 4d. The intensity of the peaks characteristic of the epoxy ring at 970–901 cm^−1^ (stretching C–O of oxirane group) and 708–781 cm^−1^ (stretching C–O–C of oxirane group) decreases significantly until disappearance, which confirms the consumption of epoxy groups during copolymerization.

### 3.2. Thermomechanical Performances Investigation by DMA

**Figure 5:**
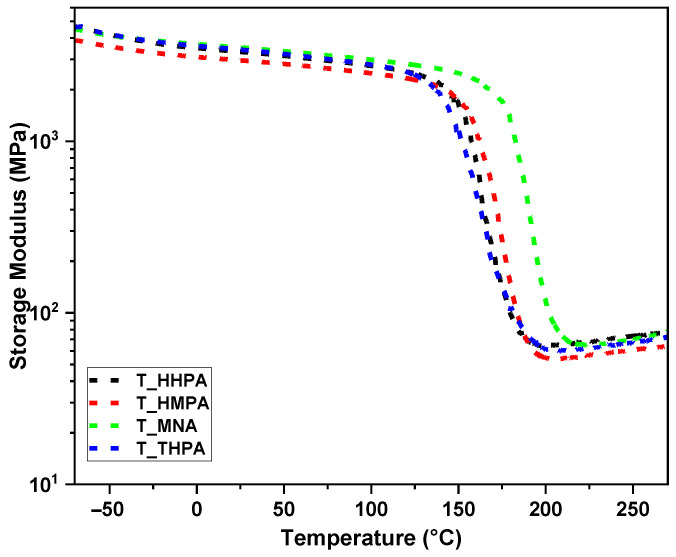
Storage moduli (*E*′) in function of temperature of designed T-anhydride thermosets.

**Figure 6:**
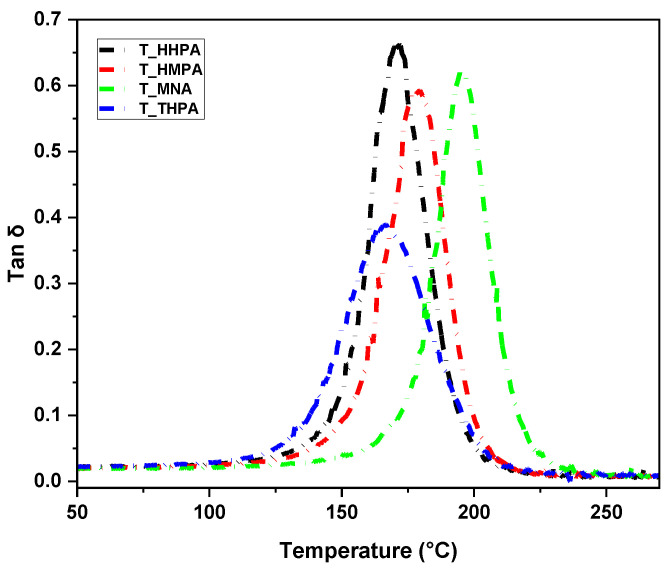
Damping factor (tan *δ*) vs. temperature curves for the epoxy-based thermoset resins.

The thermo-mechanical properties of the designed thermoset resins were investigated by DMA using a three-point bending fixture. The temperature influence on the stiffening and damping properties of the cured materials are displayed in Figure 5 and Figure 6, and the corresponding thermomechanical parameters are shown in Table 3. 

The designed thermosets show good mechanical properties revealed by high storage moduli > 3 GPa at room temperature. The highest stiffness in the glassy plateau is given by the system crosslinked with MNA with an *E*′ value of 3.5 GPa, closely followed by the THPA-system (*E*′ = 3.4 GPa) and HHPA-system (*E*′ = 3.3 GPa), the lowest storage modulus belonging to the HMPA-resin (*E*′ = 3 GPa). Compared with the previous reported studies (*E*′ = 2.8–3.1 GPa for TGPh systems and *E*′ = 2.7–3 GPa for DGEVA systems) [11], the THPMTGE-based thermoset resins revealed better mechanical properties, having a better stiffness (*E*′ = 3–3.5 GPa).

As the temperature increases, the storage modulus of the materials begins to decrease constantly until it reaches the plateau level, called the rubbery plateau. This phenomenon is determined by the increase of the free volume of the polymer chains with the thermal expansion. Knowing that the storage modulus in the rubbery region is directly proportional with the crosslink density (*ν*) of the polymeric network, the rubbery modulus was used to calculate *ν* parameter according to rubber-elasticity theory [38,39]:

(3) $$\upsilon =\frac{E^{\prime }}{3RT}$$

where *E*′ is the rubbery modulus, *R* the gas constant, and *T* the absolute temperature. Based on reported studies [40,41], *E*′ was taken at *T*_g_ + 90 °C. 

Similarly, another important network parameter such as the mass between crosslinks (*M_c_*) was determined by the Tobolsky [42] equation:

(4) $$Mc=\frac{3\rho RT}{E^{\prime }}\;$$

where *ρ* is the calculated density (g·cm^−3^), *E*′ the storage modulus in the rubbery plateau (MPa), *R* the gas constant (J·mol^−1^·K^−1^), and *T* the absolute temperature (K). The obtained results (Table 3) show that by varying the nature of the anhydride it is easy to control and modify the crosslinking density or average mass of the segments between crosslinks, thus modulating the mechanical properties of the thermosets to fit into the desired application sector. The highest stiffness was obtained by the thermoset copolymer with MNA, with a crosslink density value of about 6.4 mmol∙cm^−3^, while the crosslinking of tris-epoxide with HMPA led to a thermoset resin with a lower crosslink density (~4.8 mmol∙cm^−3^) by about 26% compared to the previous one. Also, the width of the damping peaks is consistent with the calculated crosslink densities. 

A critical indicator determining the material working temperature, which depends mainly on the rigidity of the network and the crosslink density, is the glass transition (*T*_g_). The *T*_g_ of the materials is a dynamic phenomenon directly dependent on various physico-chemical and mechanical factors [43,44]. The glass transitions (*T*_g_) of the designed thermosets were recorded by DMA at the maximum temperature of the damping factor (tan *δ* peak), while by DSC, the *T*_g_ values were registered as the inflection point of the thermograms in the second heating scan. 

The trifunctional THPMTGE monomer led to the development of compact high-crosslinked networks with small segment chains between crosslinks to materials with high mechanical properties. In addition, the variation of the chemical structure of the comonomers allowed a flexible adjustment of the final properties to satisfy different application requirements. Thus, according to the tan *δ* curves plotted in Figure 6, the softer material is the thermoset T-THPA, with a maximum of tan *δ* of about 167 °C. Depending on the nature of the anhydride, the thermosets’ glass transition increases considerably, reaching values of up to ~196 °C for the T-MNA system. The obtained thermo-mechanical results show that the synthetized thermosets fulfill the industrial criteria and requirements to be integrated and used in the automotive or aerospace sectors where hard materials resistant to extreme factors are required [7,45,46]. Glass transition values similar to those of the thermosets developed in this study are specified for commercial resins such as Solvay Cycom^®^ 977-3 (*T*_g_ = 178–190 °C), Dow Voraforce™ TW 103/TW 158 (*T*_g_ = 175–185 °C), Henkel Hysol Benzoxazine 99110 (*T*_g_ = 161–191 °C), Hexcel^®^ HexPly^®^ 8552 (*T*_g_ = 154–200 °C), Park Aerospace Nelco^®^ N4000-29 (*T*_g_ = 185 °C), etc. (based on MatWeb database), but also to the TGPh-based resins (*T*_g_ = 178–211 °C) [11], and superior to DGEVA-based resins (*T*_g_ = 93–107 °C) synthetized in the previous work [11] or to DGEBA-based resins (*T*_g_ = 46–155 °C) [35,47,48,49]. 

The surface hardness of the materials was also tested and analyzed by Shore hardness tests. The obtained result ranged between 84 and 90 Shore D being superior to previously developed materials (TGPH systems = 87–89 SD, DGEVA-systems = 80–81 SD) [11]. The developed thermosets can be classified as extra hard materials being similar to commercial ones: Kohesi Bond KB 1427 HT-3 = 85 SD; Henkel Loctite^®^ Stycast^®^ EO 1058 = 90 SD, etc.

### 3.3. Tensile Tests

Mechanical properties such as tensile strength, elongation at break and Young’s modulus of the designed thermosets were studied and determined by tensile tests for a better and more detailed characterization of the materials to facilitate their inclusion in the targeted areas of application. 

According to the obtained experimental data (Table 4), the designed resins reveal a rigid character, while the anhydride nature slightly influences the mechanical properties of the final network. The highest absolute value of tensile stress is that of T-HHPA resin 45 MPa, followed by T-HMPA and T-MNA with 44 and 41 MPa, respectively. The lowest value of about 34 MPa was measured for T-THPA. In terms of elongation at break, the resins follow the same order, except that the main place is taken by the system T- HMPA 5.1%, and the T-HHPA resin falling on the secondary place with an elongation at break of about 4.8%. The relatively low values of elongation at break obtained in this study denote the development of rigid materials, a fact already confirmed by the values of glass transitions and Shore D hardness.

Young’s modulus describes the rigidity of a material, and the value of this parameter is given by the relationship between stress and deformation in the regime of linear elasticity of uniaxial deformation. Based on the obtained data, the highest stiffness value was obtained for the T-MNA thermoset, with a value of 1.31 GPa, followed by a T-THPA of 1.29 GPa. The lowest values of the Young’s moduli of ~1.25 GPa were obtained for the T-HHPA and T-HMPA thermosets. Thus, the crosslinking of commercial tris-epoxy with the four potential biobased anhydrides led to the development of rigid materials with tensile parameters comparable with the commercial ones used in aerospace, optical, electronic, and cryogenic applications where reliable and robust materials are required: Master Bond EP51ND-tensile strength ~34.5–41.4 MPa and *E* ~1.03–1.38 GPa; Henkel Loctite^®^ Eccobond EO7021-tensile strength ~48 MPa and *E* ~1.45 GPa; Hybrid Plastics POSS^®^ EP 2000 having *E* ~1–1.50 GPa; DSM Somos^®^ 8120-tensile strength ~26 MPa and *E* ~0.276–0.703 GPa.

A significant mechanical parameter used in the materials design is the brittleness (*B*) which, according to the studies developed by W. Brostow et al. [50,51,52], can be calculated using the equation:

(5) $$B=1/\varepsilon_{b}E^{\prime }$$

where *ε_b_* represents the elongation at break obtained by tensile testing and *E*′ is the storage modulus value obtained by DMA analysis at room temperature. The brittleness parameter is inversely proportional to the *E*′ modulus at a frequency of 1.0 Hz. Therefore, the low value of the brittleness shows a dimensional stability of the materials and thus a rigid character. The calculated values of the specific modulus, specific strength and specific lengths are tabulated in Table 4. The specific modulus and specific strength are distinct characteristics that are important in the design of efficient and safe structures. The specific modulus is the property of the materials consisting of its elastic modulus per mass density, while the specific strength is its strength divided by its own density. High values of both parameters are required in space and aerospace applications where a maximum strength is needed for a minimum weight (to save weight without sacrificing resistance). The materials developed in this work reveal that the values of specific modulus ranged between 1.07–1.16·10^6^ m^2^/s^2^, these values being slowly inferior to the commercial epoxy matrices used in aerospace/ space area (Hexcel HexPly M18 = 3.01·10^6^ m^2^/s^2^, Hexcel HexPly M36 = 2.99·10^6^ m^2^/s^2^, Hexcel HexPly 108 = 2.81·10^6^ m^2^/s^2^, Hexcel HexPly EH25 = 2.78·10^6^ m^2^/s^2^). The highest specific stiffness values are achieved by materials such C diamond (347·10^6^ m^2^/s^2^) and Dupont E130 carbon fiber (417·10^6^ m^2^/s^2^). In terms of specific strength, the prepared thermosets have values between 29–40 kN·m/kg, and are thus comparable to the epoxies used in high-end industries such as aerospace and space such as Hexcel HexPly 914 (36.8 kN·m/kg), Hexcel HexPly 922-1 (44.3 kN·m/kg), or Hexcel HexPly 108 (46.8 kN·m/kg). Another way to describe the specific strength of materials is their breaking length, also known as self-support length (*L_s_*). This feature represents the maximum length of a suspended vertical column of material (supported only at the top) generated by its own weight, and is calculated based on the equation [53]:

(6) $$L_{s}=\frac{\sigma /\rho }{g}$$

where *L_s_* is the length, *σ* is the tensile strength, *ρ* is the density and *g* is the acceleration due to gravity (~9.8 m/s^2^). The *L_s_* values range between 2.99–4.06 km (commercial epoxies: Hexcel HexPly 914 = 3.77 km, Hexcel HexPly 922-1 = 4.52 km, Hexcel HexPly 108 = 4.78 km) confirming once again the good mechanical properties of these thermosets and supporting their potential use on in the aeronautics, space and automotive sectors

### 3.4. Thermal Stability of the Crosslinked Epoxy Resins

**Figure 7:**
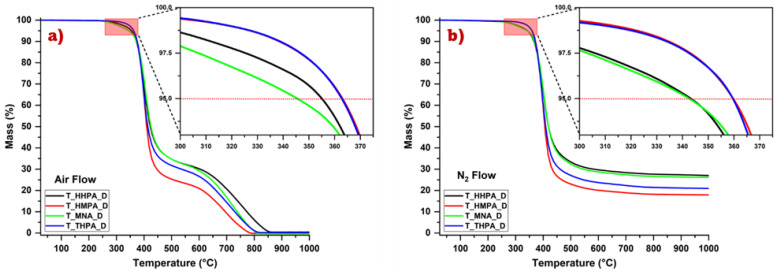
TGA thermograms obtained during heating the thermosets at 10 °C/min under (**a**) air flow, and (**b**) N_2_ flow.

The thermal behaviour of the developed epoxy resins was investigated by thermogravimetric analyses under both oxidative and inert atmospheres. The obtained mass loss (TGA) and mass derivative (DTG) thermograms are presented in Figure 7 and Figure S5, respectively. 

The initial decomposition temperature of the materials was selected as the temperature at which the systems lost 5 wt.% (*T*_5%_), while the maximum degradation temperature was related to the value of the maximum peak of the derivative of the mass lost (*T*_dmax_). These two parameters together with the temperature at which the materials lose 30% of the mass (*T*_30%_) were used for the calculation of the statistic heat resistance index *T*_s_ as well as of the char yield at 800 °C (*C*_*y*800_(%) Table 5). The statistic heat resistance index *T*_s_ is related to the physical heat tolerance limit temperature of the materials and can be calculated based on the equation [30,54]:

(7) $$T_{s}=0.49\left[T_{5\%}+0.6\left(T_{30\%}-T_{5\%}\right)\right]$$

The calculated *T_s_* values (Table 5) of the studied thermosets are similar for both oxidative and inert atmospheres, and ranged between 183–188 °C. Based on the industrial criteria, the designed polymeric materials are classified in the second (from fourth) category of the heat-resistant materials (*T_s_*: 100~200 °C) [55].

According to Figure 7 and Table 5, the developed materials exhibit a high degradation temperature in both atmospheres, their *T*_5%_ ranging from 340–360 °C. These results are comparable to the commercial ones, as, for example, Park Aerospace Nelco^®^ N4350-13 RF: *T*_5%_ = 350 °C and Park Aerospace Nelco^®^ N4000-6 FC: *T*_5%_ = 325 °C).

In an air atmosphere, the thermal behaviour of the crosslinked resins is depicted by two principal steps of decomposition. The first stage ranges from 230 °C and 550 °C, with a maximum thermal decomposition of about 400 °C. The mass loss associated to this stage is characterized by the thermolysis of the network structure. As can be seen, the lowest mass loss in this first step corresponds to T-HHPA resin (~67%), and the highest is attributed to T-HMPA thermoset (~75%), these results being consistent with their crosslink density, as higher the crosslinking density, the lower the mass loss. The second stage of degradation takes place between 560–840 °C with a maximum at about 700–770 °C. In this second thermal stage, the thermosets have approximately 25–30% mass loss, this process being associated with the thermo-oxidative degradations of the sample [56]. 

In Table 5 the char yield at 800 °C (*C_y_*_800_) was reported for both air and nitrogen atmospheres, respectively. It is possible to observe the presence of a large amount of carbon residues in nitrogen ~18–28%, a value indicating that the thermosets have good flame retardancy properties. It is known that the formation of carbon residues in nitrogen can improve the flame resistance of thermosets, the formed layer thus acting as a protective barrier that can reduce the transfer of heat and oxygen, preventing the spread of fire. [57,58] A parameter reflecting fire retardancy is the Limiting Oxygen Index (*LOI*), which can be evaluated from the char yield (*C_y_*_800_) [58,59].

(8) $$LOI=17.5+0.4\times C_{y800}$$

where *C_y_*_800_ is the mass percentage of char residue at 800 °C under N_2_ flow.

The calculated *LOI* values of the designed thermosets range between 25–29%, the highest value being obtained for T-HHPA. Based on the reported results, materials with *LOI* > 28% are classified as “self-extinguishing” systems, as in the case of the T-HHPA and T-MNA thermosets.

Thus, the designed thermosets have high thermal stability and good flame-retardant potential, which are required properties for applications in the aerospace area or electrical engineering.

### 3.5. Moisture Behaviour of the Designed Epoxy Thermosets

The polymers water absorption can lead to various changes, either reversible or irreversible, in the materials properties that could affect their performances. The moisture behaviour of the designed systems was analyzed in accordance with ASTM D570 standard and the evolution in time is graphically reported in Figure S6. The absorbed water values after 24 h of immersion and at saturation stage after about 70 days of immersion were recorded at room temperature and presented in Table 6. 

The ability of a material to absorb water can be influenced by a multitude of factors, both physical and chemical. For example, the crosslinking protocol and parameters, as well as the presence of microcracks, can facilitate the water immersion in the depth of the material. Also, chemical factors such as the presence of polar groups and free hydroxyls influence the materials’ hydrostability due to the strong water-polymer interactions (hydrogen bonds) [60,61,62,63,64]. In Figure S6, we can observe in the first time interval an increase in mass at a high rate of absorption, associated with a Fickian process which is characterized by an increase in mass to an equilibrium value which then remains constant in time [65]. We can generally observe a good *WA*% behavior of the produced thermosets. The water uptake value after 24 h of immersion at room temperature ranges between 0.32–0.48%, the *WA*% increasing in the order T-MNA < T-HMPA < T-THPA < T-HHPA. Although the developed systems had a quick increase of the *WA*% in 24 h, after 70 days of immersion the resins reach the saturation stage at low values of *WA* < 1% (~0.77–0.96%). The water absorption behavior of the designed materials is in accordance with their gel content (Table 2); the higher the gel content, the more crosslinked the networks and the lower the water sorption. 

Analyzing the obtained results, we can conclude that the developed systems have a very good hydrostability, being superior to the epoxy resins previously synthetized (TGPH-systems = 1.75–2.8% *WA*, DGEVA-systems = 1.5–1.75% *WA*, after 15 days) [11] or those reported in the literature (*WA* ~1–9%) [36,60,66], and comparable to the commercial ones currently used in aerospace and electronics sectors: e.g., Henkel Loctite^®^ Ablestik 2053S Epoxy *WA* ~1%; Hexcel^®^ F161 Epoxy Resin *WA* ~2.8%; Cookson Group Staychip^®^ 3100 *WA* ~1.14%.

## 4. Conclusions

The starting point for this study was the growing need for new and sustainable epoxy thermosets for the development of high performance materials for applications in the automotive, construction, naval, space or aerospace industries. Starting from an epoxy compound such as tris(4-hydroxyphenyl)methane triglycidyl ether, which is generally used in cosmetics, paints and coatings, or inks for printing and writing, new thermoset resins were developed by its copolymerization with four potential biobased anhydrides. In the first step, the uncured mixtures were analyzed by ATR FTIR in temperature to investigate the structural evolution of the systems, corroborated with dynamic DSC studies to follow the thermal reactivity and to establish the proper parameters of curing and post-curing. The designed formulations revealed a high reactivity, with an onset temperature of approximately 55–75 °C and a maximum temperature of reaction at about 140 °C, the obtained resins showing a very good crosslinking by *GC* values > 99.9%. The thermosets’ thermo-mechanical properties investigated by DMA analyses reveal the development of very hard materials with *E*′ ~3–3.5 GPa, and a maximum of tan *δ* which ranged between 167 and 196 °C. The thermosets have also high values of Shore D hardness ~84–90 SD. The thermosets’ rigidity was confirmed by Young’s moduli values ranging between 1.25–1.31 GPa, an elongation at break of about 4–5% and a tensile stress of ~35–45 MPa. The TGA analyses also show that the prepared resins have a very good thermal stability, superior to 340 °C, and a statistic heat resistance index ~190 °C. The *LOI* parameter was also evaluated and calculated showing the development of new materials with good flame retardancy properties. All of the four systems revealed a low hydrophilicity, the *WA* at saturation level (after 70 day of immersion) ranging from ~0.77–0.96%.

Therefore, we can conclude that thermoset materials were developed, using commercial, non-toxic compounds with a high biobased potential in this study through a fast and feasible industrial process, and with very good mechanical properties similar or even superior to those already used. All of these considerations make these thermosets potential candidates for their use in everyday applications for electronic equipment like phones, computers, cameras, or related electronic components (motherboards, software, chargers) or as high performance materials for the aerospace and space sectors.

## Figures and Tables

**Table 1:** Component amounts for 100 g of thermosetting resin.

Sample	Quantity (g)					
	THPMTGE	D	HHPA	HMPA	MNA	THPA
T-HHPA	66	1	33	-	-	-
T-HMPA	64	1	-	35	-	-
T-MNA	63	1	-	-	36	-
T-THPA	66	1	-	-	-	33

**Table 2:** DSC data of the epoxy-anhydride crosslinking and *GC*% obtained by solvent extraction method.

Sample	*T*_peak_ (°C)	*T*_onset_-*T*_end_ (°C)	Δ*H* (J·g^−1^)	*GC* (%)
T-HHPA	138	60–180	341	99.91
T-HMPA	134	55–200	329	99.95
T-THPA	137	75–190	325	99.90
T-MNA	146	95–240	278	99.92

**Table 3:** Thermo-mechanical and network properties of the designed thermosets.

Thermoset	Density (g/cm^3^)	Shore D	*E*′ at 25 °C (GPa)	*E*′ at 200 °C (GPa)	tan *δ* (°C)	*T*_g-DSC_ (°C)	*ν* (mmol∙cm^−3^)	*M*c (g/mol)	Sample Aspect
T-HHPA	1.13	88	3.3	0.08	171	179	5.70	204.43	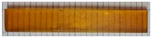
T-HMPA	1.17	90	3	0.07	179	167	4.74	248.01	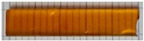
T-THPA	1.13	85	3.5	0.11	196	181	6.34	220.44	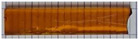
T-MNA	1.16	84	3.4	0.08	167	160	5.21	230.73	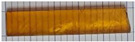

**Table 4:** Mechanical properties of the designed epoxy thermosets.

Sample	Young’s Modulus (GPa)	Tensile Stress at Break (MPa)	Elongation at Break (%)	Specific Modulus E/*ρ* (10^6^·m^2^/s^2^)	Specific Strength *σ*/*ρ* (kN·m/kg)	Specific Length *σ*/*ρ*·g (km)	*B* (%·Pa/10^10^)
T-HHPA	1.25 ± 0.16	45 ± 11	4.8 ± 1.12	1.11	39.82	4.06	0.063
T-HMPA	1.25 ± 0.15	44 ± 11	5.1 ± 1.51	1.07	37.61	3.84	0.065
T-THPA	1.31 ± 0.18	41 ± 12	4.2 ± 1.42	1.16	36.28	3.70	0.068
T-MNA	1.29 ± 0.22	34 ± 27	3.9 ± 2.77	1.11	29.31	2.99	0.075

**Table 5:** Thermal properties of the epoxy-based thermosets.

Samples	*T*_1%_ (°C)		*T*_5%_ (°C)		*T*_30%_ (°C)		*T*_dmax_ (°C)		*T_s_*		*C*_*y*800_ (%)		*LOI* (%)	
	Air	N_2_	Air	N_2_	Air	N_2_	Air	N_2_	Air	N_2_	Air	N_2_	Air	N_2_
T-HHPA	291	261	350	345	399	395	399	399	186	184	6	28	N/A	28.7
T-HMPA	320	310	360	359	395	395	399	399	188	187	0	18	N/A	24.7
T-MNA	280	261	340	340	400	395	409	401	187	183	0.7	27	N/A	28.3
T-THPA	320	310	360	360	395	395	399	395	188	187	1	22	N/A	26.3

**Table 6:** Water absorption after 24 h and at saturation for the designed thermosets.

Samples	Water Absorption (%)	
	at 24 h	at Saturation
T-HHPA	0.48	0.90
T-HMPA	0.39	0.77
T-THPA	0.32	0.84
T-MNA	0.40	0.96

## Data Availability

Not applicable.

## Supplementary Materials

The following supporting information can be downloaded at: https://www.mdpi.com/article/10.3390/polym14245473/s1, Figure S1: DSC thermograms of curing the T-MNA formulation initiated by 0.5 wt.%, 1 wt.% and 2 wt.% 2,4,6-tris(dimethylaminomethyl)phenol; Figure S2: Glass transition of the cured systems obtained by DSC; Figure S3: FTIR spectra of raw compounds; Figure S4: FTIR spectra of crosslinked thermosets; Figure S5: DTG curves of the cured materials under (c) air and (d) N_2_ Flow; Figure S6: WA% evolution with the time.
