# Supplementary material for: Development of Sustainable High Performance Epoxy Thermosets for Aerospace and Space Applications
Source: Polymers (Basel). 2022 Dec 14;14(24):5473. doi: 10.3390/polym14245473 (PMC9785596; doi:10.3390/polym14245473)
Supplement: Supplementary file 1 [file polymers-14-05473-s001.zip › polymers-2079876-supplementary.pdf]

Supporting Information

Development of Sustainable High Performance Epoxy Thermosets for Aerospace and Space Applications

Roxana Dinu [1], Ugo Lafont [2], Olivier Damiano [3] and Alice Mija [1,*]

[1] Université Côte d'Azur, Institut de Chimie de Nice, 06108 Nice, France
[2] ESA, ESTEC, Keplerlaan 1, P.O. Box 299, NL-2200 AG Noordwijk, The Netherlands
[3] Thales Alenia Space, 5 Allée des Gabians, 06156 Cannes la Bocca, France
* Correspondence: alice.mija@univ-cotedazur.fr

Total number of pages : 4

Total number of figures : 6

1. Differential scanning calorimetry

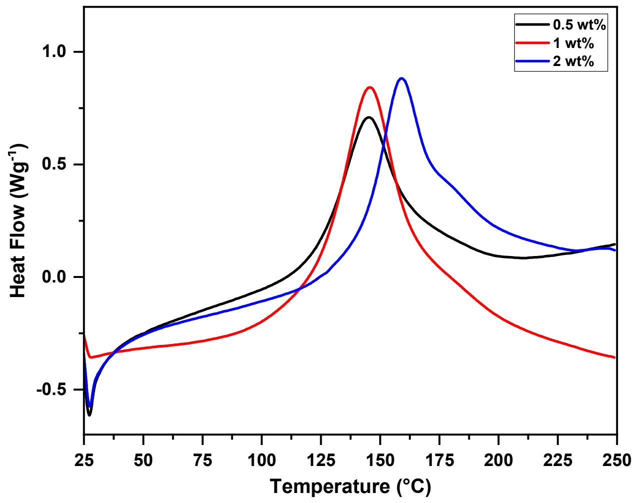

Figure S1. Dynamic DSC thermograms of curing the T-MNA formulation initiated by various percentage of 2,4,6-tris(dimethylaminomethyl)phenol: 0.5 wt.% (black line), 1 wt.% (red line), and 2 wt.% (blue line)

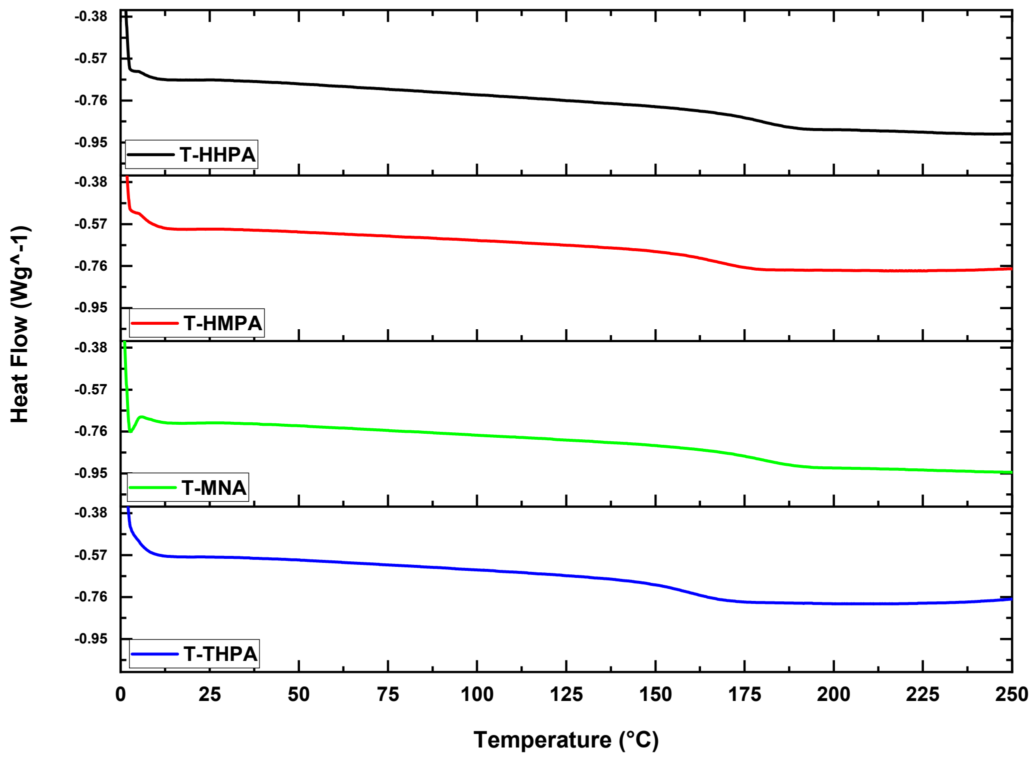

Figure S2. Glass transition of the cured systems obtained by DSC

2. FT-IR Analysis

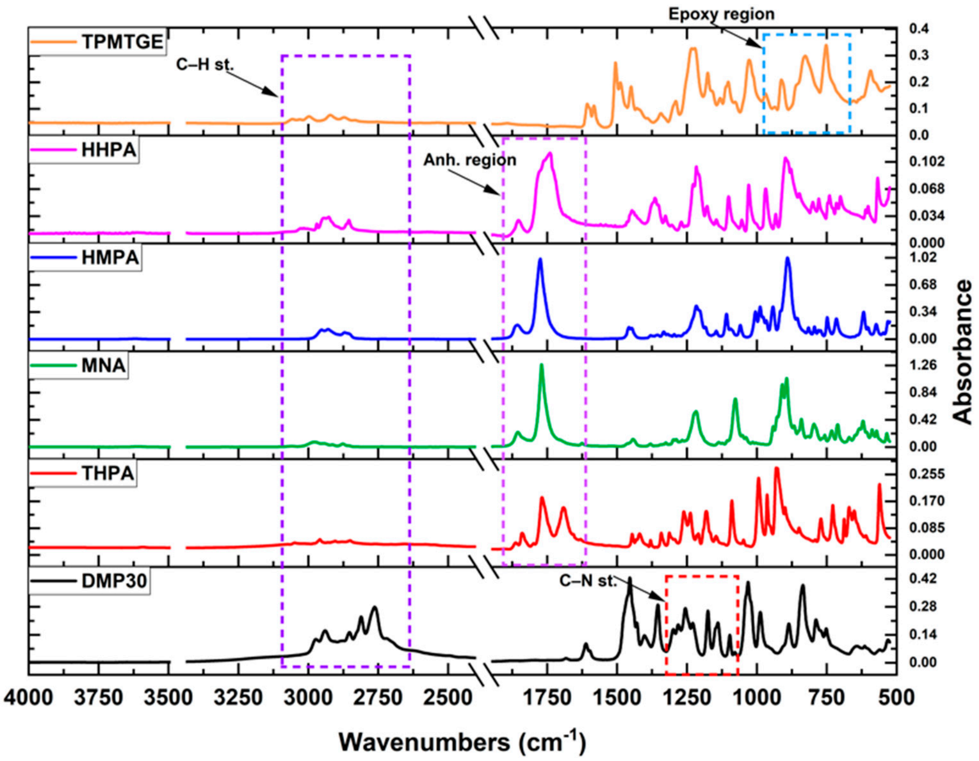

Figure S3. FT-IR spectra of raw materials

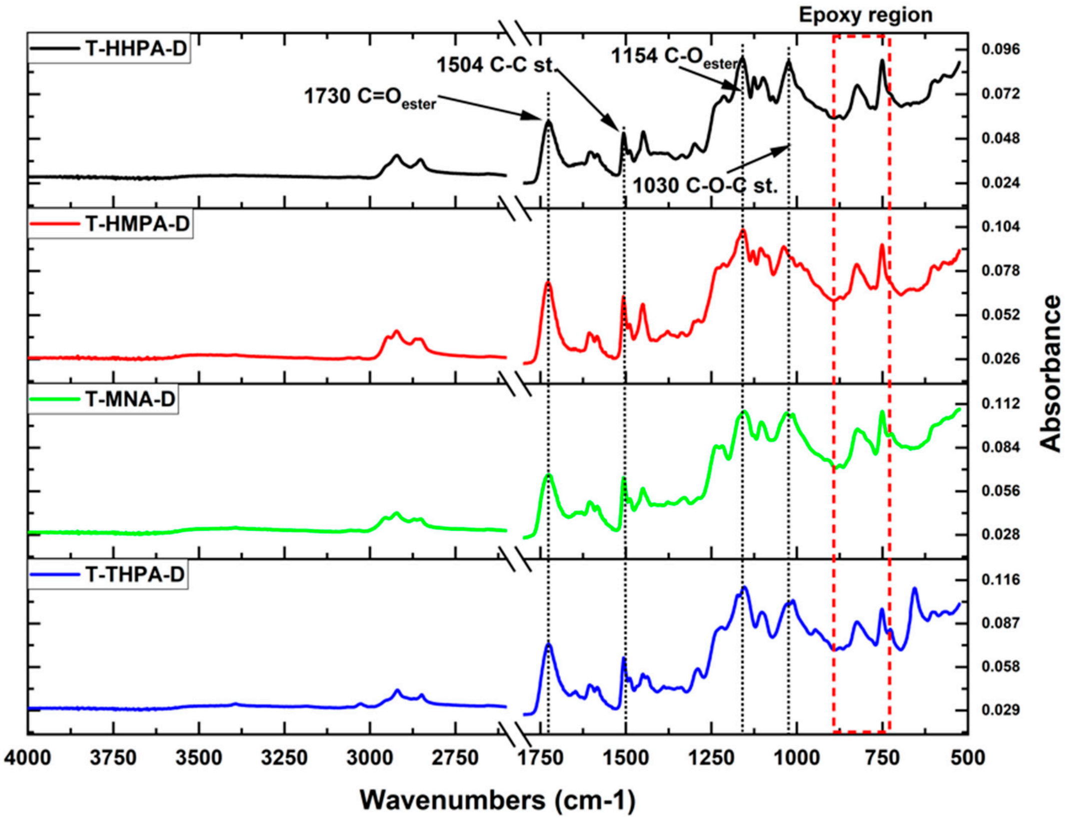

Figure S4. FT-IR spectra of crosslinked thermoset resins

2. Thermogravimetric analysis

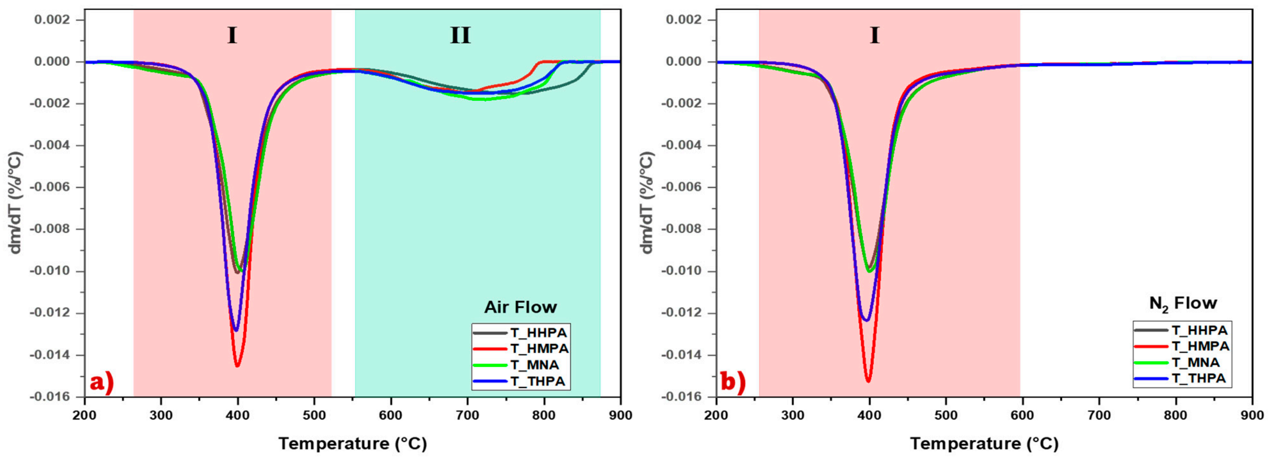

Figure S5. DTG curves of the cured materials under c) air and d) N_2 Flow

3. Moisture behaviour

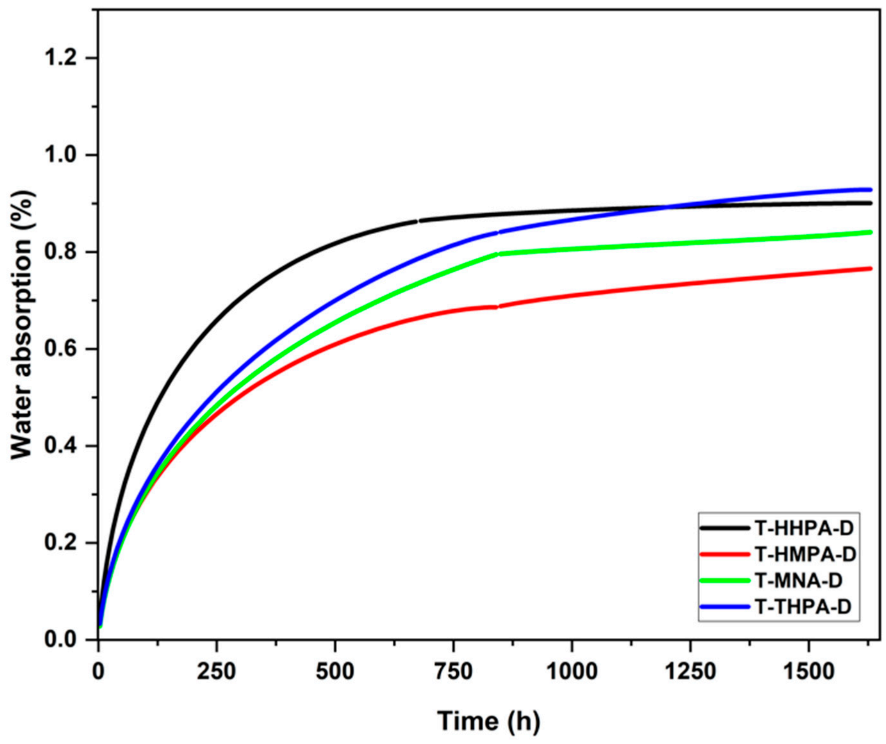

Figure S6. WA% curves of epoxy/anhydride thermosets
